# Systemic thioridazine in combination with dicloxacillin against early aortic graft infections caused by *Staphylococcus aureus* in a porcine model: *In vivo* results do not reproduce the *in vitro* synergistic activity

**DOI:** 10.1371/journal.pone.0173362

**Published:** 2017-03-09

**Authors:** Michael Stenger, Carsten Behr-Rasmussen, Kasper Klein, Rasmus B. Grønnemose, Thomas Emil Andersen, Janne K. Klitgaard, Hans Jørn Kolmos, Jes S. Lindholt

**Affiliations:** 1 Research Unit of Clinical Microbiology, University of Southern Denmark, Odense, Denmark; 2 Department of Cardiothoracic and Vascular Surgery, Odense University Hospital, Odense, Denmark; 3 Department of Vascular Surgery, Viborg Hospital, Viborg, Denmark; 4 Department of Biochemistry and Molecular Biology, University of Southern Denmark, Odense, Denmark; Universiteit Gent, BELGIUM

## Abstract

**Introduction:**

Conservative treatment solutions against aortic prosthetic vascular graft infection (APVGI) for inoperable patients are limited. The combination of antibiotics with antibacterial helper compounds, such as the neuroleptic drug thioridazine (TDZ), should be explored.

**Aim:**

To investigate the efficacy of conservative systemic treatment with dicloxacillin (DCX) in combination with TDZ (DCX+TDZ), compared to DCX alone, against early APVGI caused by methicillin-sensitive *Staphylococcus aureus* (MSSA) in a porcine model.

**Methods:**

The synergism of DCX+TDZ against MSSA was initially assessed *in vitro* by viability assay. Thereafter, thirty-two pigs had polyester grafts implanted in the infrarenal aorta, followed by inoculation with 10^6^ CFU of MSSA, and were randomly administered oral systemic treatment with either 1) DCX or 2) DCX+TDZ. Treatment was initiated one week postoperatively and continued for a further 21 days. Weight, temperature, and blood samples were collected at predefined intervals. By termination, bacterial quantities from the graft surface, graft material, and perigraft tissue were obtained.

**Results:**

Despite *in vitro* synergism, the porcine experiment revealed no statistical differences for bacteriological endpoints between the two treatment groups, and none of the treatments eradicated the APVGI. Accordingly, the mixed model analyses of weight, temperature, and blood samples revealed no statistical differences.

**Conclusion:**

Conservative systemic treatment with DCX+TDZ did not reproduce *in vitro* results against APVGI caused by MSSA in this porcine model. However, unexpected severe adverse effects related to the planned dose of TDZ required a considerable reduction to the administered dose of TDZ, which may have compromised the results.

## Introduction

Aortic prosthetic vascular graft infection (APVGI) is a serious and therapeutically challenging complication. In earlier series, mortality and lower-limb amputation rates are reportedly up to 88% and 25%, respectively [1–3]. Methicillin-sensitive *Staphylococcus aureus* (MSSA) is the most frequently isolated bacteria in the early phase (≤4 months) of APVGIs, accounting for 43–64% of the isolates [4, 5]. New treatment regimens involving complete *in situ* graft replacement with an autogenous conduit or allograft have lowered mortality and reinfection rates considerably [1, 6]. Nonetheless, patients with APVGIs are often elderly with comorbidities, who may not be eligible for open surgery. Furthermore, an increasing number of inoperable patients are treated with endovascular aortic repair (EVAR), and infections related to endoprosthetic grafts in this category of patients render them less favourable for open surgery. Consequently, conservative treatment with antibiotic only and complete graft preservation in high risk patients has been evaluated with limited rates of success [7–10]. The insufficient effect of conservative APVGI treatment is probably a combination of low antibiotic bioavailability at the infection site and the formation of a protective biofilm layer, which significantly reduces the susceptibility of the microorganism [11, 12]. The use of a helper compound, such as the antipsychotic drug thioridazine (TDZ), can significantly potentiate the antibacterial efficacy of β-lactam antibiotics against both methicillin-resistant and susceptible *S*. *aureus* strains and induces a synergetic effect *in vitro* [13–16]. On the other hand, conflicting results have been obtained in animal studies comprising *C*. *elegans* and mice [17–19]. The antibacterial mechanisms of TDZ are not completely understood, but it is known to induce major changes in transcription of genes related to pathways in cell wall biosynthesis [20, 21]. Furthermore, static *in vitro* experiments indicate that TDZ reduces staphylococcal biofilm formation by inactivation of efflux pumps [22] and enhanced loading capacity of DCX when co-loaded with TDZ into a polymer hybrid silicone material [23], making the combination of TDZ and a β-lactam antibiotic a particularly interesting option for the management of APVGIs caused by *S*. *aureus*.

The aim of this study was to investigate the efficacy of conservative systemic treatment of TDZ in combination with dicloxacillin (DCX), compared to DCX alone, against early APVGI caused by MSSA in a porcine model. Initially, we determined the *in vitro* efficacy of combining DCX and TDZ against the MSSA test strain. The *in vivo* efficacy was evaluated primarily in terms of bacteriological endpoints and secondarily by changes in weight, temperature, white blood cell count (WBC), haemoglobin, and haptoglobin. Finally, we report on observations concerning animal welfare, treatment compliance, and adverse drug effects.

## Materials and methods

### Antimicrobial agents

For the *in vitro* experiments we used DCX (Sigma-Aldrich Corporation, Denmark) and TDZ (thioridazine hydrochloride, Sigma-Aldrich Corporation, Denmark) in its racemic form. For the *in vivo* experiments DCX capsules (Dicillin® 250 mg; Sandoz, Denmark) were purchased and used as commercially registered in Denmark for clinical use [24]. The purchase of TDZ tablets (Thioridazin-neuraxpharm® 25 mg; thioridazine hydrochloride, Neuraxpharm, Germany) was licenced by the Danish Health and Medicines Authority (reference no.: 2013041542).

### Bacterial test strain

Throughout this study, MSSA (ATCC 29213) was utilized. This strain had previously been used and validated by Gao et al. in a similar porcine model [25–27]. The strain was susceptible to oxacillin, gentamicin, ciprofloxacin, rifampicin, vancomycin and cefoxitin, but resistant to penicillin.

### MIC, MBC and viability assay

Minimal inhibitory concentration (MIC) and minimum bactericidal concentration (MBC) tests were respectively performed for DCX and TDZ according to Clinical Laboratory Standards Institute guidelines, and effects on bacterial growth of DCX and TDZ alone and in combination were tested by a viability assay as previously reported by Klitgaard and colleagues [13].

### Animals

We used domestic female pigs from a specific-pathogen-free herd at the Department of Animal Science, Aarhus University. All pigs were approximately three and half months old at the beginning of the experiment. During the entire study period the pigs were housed individually in pens with saw-dusted solid floors and drinking nipples. They were fed three times daily. All pigs were acclimatized under these conditions for at least one week before initiation of the experiment. The animal study protocol was approved by the Danish Animal Experimentation Inspectorate (license no. 2012-15-2934-00290).

### The APVGI porcine model

The APVGI porcine model was carried out as a part of a tandem project performed in the same series of pigs involving a central venous catheter infection model [23]. Inoculation and implantation of the aortic grafts and central venous catheters were performed during the same operation using the same MSSA strain (ATCC 29213).

The anaesthetic and surgical procedure has previously been described [25–27]. In brief, pigs were placed in a supine position and the surgical area was prepared with alcohol-based chlorhexidine and sterile dressings. All pigs were given a single dose of cefuroxime 1500 mg intravenously immediately before skin incision. The abdominal aorta was exposed through a 25–30 cm long midline laparotomy and the lumbar and inferior mesenteric arteries were clipped and cut. After an intravenous administration of 5000 IU of heparin, the aorta was clamped and the infrarenal aorta was replaced by an approximately 5 cm long collagen coated knitted polyester graft (Gelsoft 8 mm; Vascutek) by end-to-end anastomoses using 4–0 polypropylene sutures. Haemostasis was secured and the surface of the graft was directly inoculated with 10^6^ CFUs of MSSA (ATTC 29219) in 0.3 mL saline solution using a 1-mL syringe. Hereafter, the retroperitoneum and abdominal wall were closed in layers with standard surgical techniques. All pigs received analgesic treatment with intramuscular injections of flunixin 150 mg with 24-hour intervals and buprenorphine 0.6 mg with 8-hour intervals for the first three days after surgery. The day of treatment initiation was defined as baseline (day 0) and therefore the day of the operation was defined as day -7. Weight and rectal temperature were measured and blood samples were collected before the operation (day -7) and at day -6 (only temperature), -5 (not weight), 0, 7, 14, and 21. These parameters were always obtained in the morning prior to treatment administration. Healthy pigs gain approximately 0.5–1.0 kilogram per day and have a normal rectal temperature range of 38.7–39.8 Celsius [28]. The blood samples were used for analysis of white blood cell count (WBC), haemoglobin (HGB) and haptoglobin—the latter being an infection marker in pigs and equivalent to C-reactive protein (CRP) in humans. A decrease in HGB and an increase in WBC and haptoglobin were interpreted as signs of an ongoing infection. Moreover, the animal keepers recorded several other parameters on an ordinal scale, such as stool (yes/no), movement (normal/impaired/abnormal), wound appearance (no infection/superficial infection/severe infection), and treatment compliance (eaten/partially eaten/not eaten/no treatment given) on a daily basis.

### Treatment groups, dosages, and administration

Based on human equivalent dosages we initially planned to use a TDZ dosage of 300 mg x 2 daily, which was equivalent to 10 mg/kg/day in a 60 kg pig [29]. However, this had to be considerably reduced due to adverse drug effects. The final treatment regimens used are described below. The drugs were administered orally in two different treatment groups: 1) DCX (Dicillin 750 mg twice daily) and 2) DCX+TDZ (Dicillin 750 mg twice daily + Thioridazin-neuraxpharm 50 mg twice daily the first four days (day 0–4) and 100 mg twice daily for the rest of the treatment period (day 5–21). To enhance compliance the capsules/tablets were broken/crushed and mixed with dog food and regular pig food. Treatment was initiated 7 days after the implantation and inoculation of the aortic graft (day 0) and continued for a further 21 days. The pigs were consecutively allocated to the treatment groups by a fixed randomisation key (1, 2, 2, 1, 1, 2,…) and the animal keepers selected the pigs for surgery blinded from this random allocation.

### Sampling and microbiological analyses

After 21 days of treatment, the pigs were anesthetized and the aortic graft was exposed through the previous incision under aseptic conditions. A swab with a cotton stick was done from the surface of the graft and placed in a tube with 1 mL PBS + 0,1% Triton X-100 (Sigma-Aldrich Corporation, Denmark). Tissue from the perigraft area was randomly sampled and transferred to a plastic tube with 10 ml isotonic saline (Amgros, Denmark). The aorta was ligated above and beneath the aortic graft and a minimum of 3 cm of the graft was cut out and transferred to a plastic tube with 40 ml of isotonic saline. Finally, the pigs were euthanized with an intravenous injection of phenobarbital.

Immediately, the samples were transferred to the laboratory for microbiological analysis. The swab was vortexed for one minute, and the cotton stick was wiped off on the inside of the tube before removal. Under aseptic conditions, 1 cm of the graft was cut off and placed in a tube with 5 ml PBS + 0.1% Triton X-100 and vortexed for 1 min. The tissue was weighed and homogenized in 10 ml of isotonic saline. All three solutions from the swab, tissue and graft were individually plated on Mueller-Hinton agar plates in ten-fold serial dilutions for CFU determination with 0.1 ml on each plate.

### Statistical analyses

Bacterial count data from all three primary bacteriological endpoints (graft, swab, and tissue) were converted into CFU/mL and added by one (+1) to avoid zeros and allow transformation by natural logarithm (ln). Despite different transformation attempts of the data a normal distribution could not be achieved and thus, a two-sample Wilcoxon rank-sum (Mann-Whitney) test was used for comparison. Furthermore, bacterial quantity data from each bacteriological endpoint were converted into binary categorical variable and labelled: “No growth” or “Growth” referring to CFU/mL = 0 or CFU/mL>0, respectively and compared by two-sided Fisher’s exact test. Mixed models were used in the analysis of longitudinal continuous variables (weight, temperature, WBC, HGB, and haptoglobin) to pursue individual changes within treatment groups over time. Determination of the mixed model was based on the research question alone. Treatment group and time (including interaction) were set as fixed values, and baseline values (day 0) as covariates. Pig number (ID) was set as the random intercept. Ordinal outcomes of the variables: defecation, movement, wound healing, and treatment compliance were compared between treatment groups by a Chi-squared test. A p-value of <0.05 was considered significant. Stata/IC version 13.1 (Statacorp) was used for illustrations and analyses.

### Sample size calculation

Sample size of the study was set upon a power calculation to detect a 50% difference in the frequency of graft infections or a 15% difference in bacterial quantities with an 80% power and a 5% significance level, assuming that 100% of the grafts will be infected by the end of the study in pigs from the control group (DCX treatment only).

## Results

### MIC, MBC and viability assay

**Fig 1:**
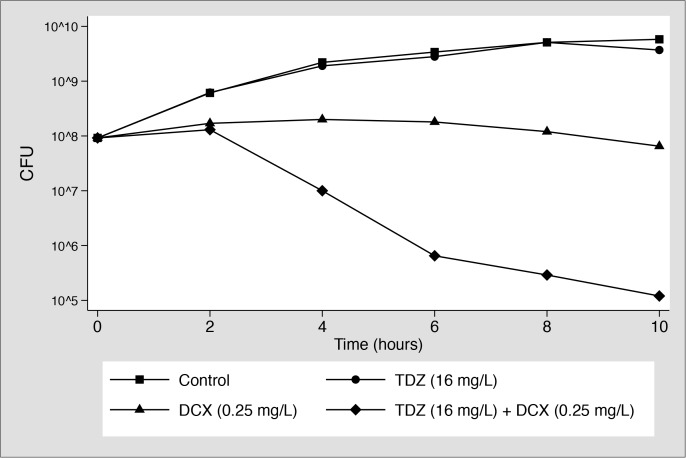
*In vitro* viability assay: The effect of thioridazine (TDZ) and dicloxacillin (DCX), alone and in combination, on survival of MSSA (ATCC 29213) over time. Control: No drug.

DCX and TDZ had MIC/MBC-values of 0.5/2 mg/L and 32/64 mg/L, respectively. Results of the *in vitro* viability assays are presented in Fig 1. In accordance with the MIC/MBC value the growth of our MSSA test strain was not inhibited by TDZ (16 mg/L) and revealed the same growth pattern as for the control (no drug). DCX (0.25 mg/L) slightly affected growth and may therefore be characterised as a sub-inhibitory concentration. However, the combination of DCX (0.25 mg/L) and TDZ (16 mg/L) showed a significant and synergetic inhibition of growth with a reduction of bacterial load of approximately 3 and 4.5 log_10_ CFU compared to DCX (0.25 mg/L) or TDZ (16 mg/L) alone, respectively, after 10 hours.

### The APVGI porcine model

According to the sample size calculation, 32 pigs were included. Seventeen pigs were allocated to the combination treatment (DCX+TDX) and 15 pigs to monotherapy (DCX). Three pigs had to be euthanized before the end of the experiment: two pigs on day 2 due to spinal cord ischemia (both allocated to the combination treatment), and one pig on day 21 due to persistent high fever (allocated to the DCX-group). In total, 29 pigs (91%) survived until the end of the experiment with a treatment group distribution of 14 pigs in the DCX-group and 15 pigs in DCX+TDZ-group. Baseline characteristics before treatment initiation (day 0) of all pigs were recorded and sorted by treatment group as presented in Table 1. Comparisons of the treatment groups revealed non-significant p-values for all parameters.

**Table 1:** Baseline characteristics, listed for all pigs and by treatment group.

Variables	All pigs (n = 29)	DCX (n = 14)	DCX+TDZ (n = 15)	p-value*
	Mean (SD)			
**Weight (kg)**	53.7 (2.4)	54.0 (2.8)	53.5 (2.0)	0.58
**Temperature (Celsius)**	39.2 (0.3)	39.2 (0.3)	39.2 (0.3)	0.75
**White blood cell count (10**^**9**^**/L)**	20.4 (5.9)	20.8 (7.1)	20.1 (4.6)	0.78
**Haemoglobin (g/dL)**	9.6 (0.7)	9.6 (0.5)	9.6 (0.8)	0.93
**Haptoglobin (mg/mL)**	3.6 (0.8)	3.4 (0.9)	3.7 (0.8)	0.38

* Comparison of the two treatment groups (DCX vs. DCX+TDZ) by t-test with equal variance at baseline (day 0) immediately before treatment initiation.

### Primary bacteriological endpoints

Following three weeks of conservative treatment with either DCX alone or DCX+TDZ in combination we determined the bacterial quantities from graft surface swabs, graft materials and perigraft tissue. As shown in Table 2, we found no statistically significant differences in bacterial quantities between the two treatment groups with respect to graft (p = 0.209), swab (p = 0.864), and tissue (p = 0.728). Some bacterial endpoints actually revealed zero bacterial counts, and therefore we decided to transform the continuous count data into binary outcomes of growth vs. no growth for a post hoc analysis. However, we still observed no significant difference between the treatment groups. Some of the microbiological analyses were inconclusive due to contamination of the agar plates and were excluded from the analysis. The number of included observations (N) from each treatment group is displayed in Table 2.

**Table 2:** Primary bacteriological endpoints.

Variables	N (DCX/DCX+TDZ)	DCX (n = 14)	DCX+TDZ(n = 15)	*P-value*
**Graft**				
Mean (SD)	12/13	10.2 (5.9)	8.5 (5.6)	*0*.*209*^*a*^
Growth / No growth	12/13	10/2	10/3	*>0*.*999*^*b*^
**Swab**				
Mean (SD)	12/15	6.7 (3.6)	6.4 (3.3)	*0*.*864*^*a*^
Growth / No growth	12/15	10/2	12/3	*>0*.*999*^*b*^
**Tissue**				
Mean (SD)	13/15	8.8 (6.9)	8.5 (4.7)	*0*.*728*^*a*^
Growth / No growth	13/15	9/4	12/3	*0*.*67*^*b*^

^*a*^ Two-sample Wilcoxon rank-sum (Mann-Whitney) test

^b^ Two-sided Fisher’s exact test.

Bacterial quantities (ln(CFU/mL) of graft, swab and tissue presented as a mean with standard deviation (SD) and as converted binary outcomes (No growth: CFU/mL = 0 and Growth: CFU/mL > 0) sorted by treatment group. DCX: Dicloxacillin; TDZ: Thioridazine; N: Number of included observation from each treatment group.

### Secondary longitudinal continuous outcomes

**Fig 2:**
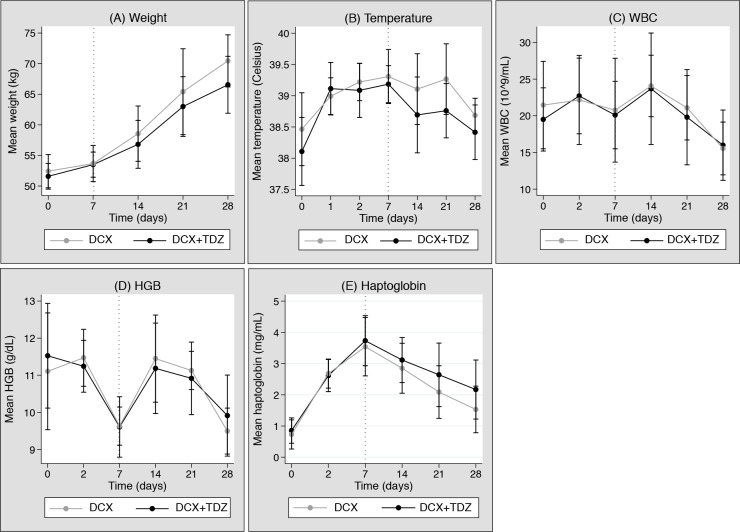
**Changes in weight **(A)**, temperature **(B)**, white blood cell count (WBC) **(C)**, haemoglobin (HGB) **(D)**, and haptoglobin **(E)** of each treatment group during the experimental period are displayed as mean values with standard deviation bars at predefined time points.** Graft implantation and inoculation was at day -7 and the dotted grey line indicates treatment initiation at day 0. Dicloxacillin (DCX); Thioridazine (TDZ)

Changes in mean weight, temperature, WBC, HGB, and haptoglobin during the experimental period for both treatment groups are illustrated in Fig 2A–2E.

Overall, the curves of the two treatment groups had similar shapes and slopes with overlapping standard deviations on all variables. However, the curves of weight, temperature, and haptoglobin slightly diverged from day 0 to day 21, meaning that the DCX-group tended to have a higher weight and temperature, but a lower level of haptoglobin than the DCX+TDZ-group toward the end of the treatment period. Furthermore, it is noteworthy that most variables improved from treatment initiation on day 0 (marked by the grey dotted line) and onwards, even though WBC increased in both treatment groups from day 0 to 7 and then decreased from day 7 to 21, and similar HGB initially improved from day 0 to 7 and then declined from day 7 to 21 (Fig 2C and Fig 2D).

Individual changes of each response variable within treatment groups over time were pursued by mixed model analysis and the coefficients are listed in Table 3.

**Table 3:** Mixed model analysis of longitudinal continuous outcomes.

Variables	Weight	Temp	WBC	HGB	Haptoglobin
	Coefficients				
**Treatment group**					
DCX+TDZ	-1.59	-0.31	-0.89	-0.28	0.20
**Time (days)**					
*Day 14*	7.32 **	0.12	-3.17 *	-0.54	-0.73 **
*Day 21*	11.34 **	-0.35*	-8.92 **	-2.24 **	-1.26 **
**Treatment#Time**					
*DCX+TDZ#Day14*	-0.99	-0.02	-1.65	0.17	0.26
*DCX+TDZ#Day21*	-0.77	0.02	1.09	0.69	0.36
**Baseline**	1.42 **	0.13	0.48 **	0.14	0.32*
**Intercept**	-17.72	34.08	14.56	10.39	1.70

Mixed model analysis of weight, temperature (Temp), white blood cell count (WBC), haemoglobin (HGB) and haptoglobin. Treatment group, time (days), and the interaction between treatment group and time (Treatment#Time) were set as fixed effects and baseline values at day 0 as covariates. Each individual pig was set as a random intercept.

*: p<0.05

**: p≤0.001).

All mixed model analyses in Table 3 are based on the DCX-group as the reference treatment group, and because no numeric conversions were made the coefficients are equivalent to the entities of each variable. The coefficients are best explained by using the response variable weight as an example. The first coefficient in the weight column explains the difference between the DCX+TDZ-group and DCX-group at the first time point after baseline, in this case day 7 (DCX+TDZ–DCX | day 7 = -1.59 kg). The next two coefficients explain the weight changes over time of the reference treatment group (DCX) compared to the measurement at day 7 and day 14, respectively (Day 14 –Day 7 | DCX = 7.32 kg; Day 21 –Day 7 | DCX = 11.34 kg. Both time points showed a highly significant increase in weight compared to day 7 (p≤0.001). The following two coefficients explain the difference between the two treatment groups at day 14 and 21 compared to the difference at day 7. Therefore, to calculate the absolute difference at day 14 and 21, the first coefficient at day 7 must be added (DCX+TDZ–DCX |day 14 = -0.99 + (-1.59) = -2.58 kg; DCX+TDZ–DCX |day 21 = -0.77 + (-1.59) = -2.36 kg.

In general, the response variables changed during the treatment period, and the last measurements on day 21 were significantly different from the first measurements after baseline (day 7) in all response variables. However, the analyses for all response variables presented no statistical significant differences between the treatment groups. The coefficients in Table 3 roughly correspond to the graphed changes in weight, temperature, WBC, HGB, and haptoglobin in Fig 2A–2E. However, they are not directly comparable since they are based on two different statistical analyses and calculations.

### Animal welfare and treatment compliance

Over the complete experimental period, the daily registrations of parameters concerning animal welfare—that is, stool (yes/no), movement (normal/impaired/abnormal), and wound appearance (no infection/superficial infection/severe infection)—included 413 observations in the DCX-group and 420 observations in the DCX-TDZ-group. The DCX+TDZ group had two observations of “no stool”, but all other observations showed daily stools with no significant difference between the treatment groups (p = 0.161). We registered 14 and 21 observations of impaired movement in the DCX- and DCX+TDZ-groups, respectively. No observations were categorized as abnormal movement in the DCX-group and only 1 in the DCX+TDZ-group. Overall, we found no significant difference in the movement parameter between the treatment groups (p = 0.310). Regarding wound appearance, signs of a superficial infection were evident in 15 and 21 observations in the DCX- and DCX+TDZ-groups, respectively. Signs of severe infection were not observed in the DCX-group and only once in the DCX+TDZ-group. Overall, the chi-squared test showed no significant difference in wound appearance between the treatment groups (p = 0.379).

Registration of treatment compliance (eaten/partially eaten/not eaten/no treatment given) during the three-week treatment period included 309 observations in the DCX-group and 315 observations in the DCX-TDZ-group. In the DCX-group, the treatment was eaten in 298/309 (96%) observations and only partially or not eaten in 8 and 3 observations, respectively. In the DCX+TDZ-group, the treatment was eaten in 296/315 (94%) observations and only partially or not eaten in 11 and 8 observations, respectively. There were no observations of “no treatment given” in any of the treatment groups. Overall, we found high treatment compliance in both groups with no statistical difference (p = 0.263).

### Adverse drug effects

Shortly after treatment initiation in the pilot study, we observed unexpected severe extrapyramidal adverse effects and behavioural changes in the pigs treated with a TDZ dose of 300 mg x 2 daily in combination with DCX. The pigs showed signs varying from dizziness and drowsiness to severe agitation with repetitive compulsive self-destructive behaviour (e.g. licking or scratching the same spot of the pen walls for several hours resulting in abrasions to the head or snout). Pigs treated with DCX alone showed no signs of these behavioural changes. In consultation with the supervisory veterinarian, we gradually reduced the dosage of TDZ until these adverse effects were acceptable in relation to animal welfare and ethical regulations. A TDZ dose reduction to less than a third of the initial dose was required to reach acceptably low levels of side effects.

## Discussion

This study is the first large-animal model investigation of TDZ used in combination with a β-lactam antibiotic as conservative systemic treatment against a vascular graft infection caused by *S*. *aureus*. Our *in vitro* experiments were able to reproduce the previously demonstrated *in vitro* synergy of DCX and TDZ against the chosen MSSA test strain, but in our porcine model the combination treatment failed to eradicate early APVGI caused by 10^6^ CFU of MSSA after three weeks of conservative systemic treatment. Based on the bacterial quantities of the three primary bacteriological endpoints, no statistical potentiated or synergetic effect of the combination treatment was found compared to treatment with DCX alone. We emphasize that both treatment groups had high treatment compliance with no statistical difference and therefore, this factor can be ruled out as a potential bias.

Before treatment initiation all pigs showed increasing temperature and haptoglobin and decreasing HBG within the first week after graft implantation and inoculation (Fig 2B, Fig 2D, and Fig 2E), indicating that APVGI was successfully and consistently established.

The bacteriological results had quite wide ranges, and a few samples within each bacteriological endpoint even showed no bacterial growth, but these findings were present in both treatment groups with no statistical difference (Table 2). Gao et al. [27], who used the same APVGI porcine model, bacterial strain and inoculum concentration, also reported large SDs in bacterial load on graft material of 2.93 (SD: 1.45) log_10_ CFU equivalent to 6.75 (SD: 3.34) ln CFU following a treatment regimen, which also failed to eradicate the graft infection. This indicates that insufficient antimicrobial treatment enhances the influence of intrinsic biological variations, which can be expressed as large SDs of quantitative bacteriological endpoints, as seen in our study.

Most secondary outcomes improved after treatment initiation, indicating some effect of both treatments, including small differences (especially for weight, temperature, and haptoglobin) between the treatment groups (Fig 2A–2E). However, the mixed model analyses of all secondary outcomes revealed no significant individual differences between treatment groups during the treatment period.

Our results are in accordance with the findings of two recent animal studies addressing the potential of systemic treatment with TDZ in combination with a β-lactam antibiotic against *S*. *aureus* [18, 19]. We have recently published assessments of TDZ as a helper compound to DCX against methicillin-resistant *S*. *aureus* (MRSA) in a modified mouse peritonitis model [18]. No synergetic effect was found using subcutaneous systemic treatment; however, increased systemic dosages of DCX+TDZ revealed an underpowered tendency towards better antimicrobial efficacy but also indications of increasing adverse effects [18]. Hahn and colleagues [19] also failed to show any synergetic effect with systemic treatment (intraperitoneal and subcutaneous administration) of TDZ in combination with cefazolin against MRSA and MSSA in a mouse skin infection model. No adverse effects were reported.

In contrast, *in vivo* synergy of TDZ+DCX against MRSA was confirmed in a *C*. *elegans* model [17] and by local intraperitoneal administration in our modified mouse peritonitis model [18]. It is noteworthy that the presence of high drug concentrations directly at the infection site is a recurrent characteristic of these two model designs, and all other animal studies reporting positive outcomes of the treatment with TDZ alone or in combination with an antibiotic [17, 18, 23, 30–33].

The promising *in vitro* results presented here and in a recently published paper [23], along with the previous report of TDZ’s anti-biofilm capacity [22], led us to this intriguing idea of TDZ in combination with a β-lactam antibiotic as a possible new conservative treatment solution against APVGI caused by MSSA. However, the *in vitro* activities did not manifest in this *in vivo* model using systemic treatment in non-toxic dosages. The reason for this discrepancy is unknown, but the following speculations could guide the design of further investigations to clarify the issue. First, we recognize that APVGI is arguably one of the most difficult infections to eradicate without supplemental surgical intervention, and so far, no valid alternative is available. Furthermore, we may have used inadequate dosages of DCX. More frequent daily dosages and intravenous administration were also initially considered, but this was not feasible under the given experimental conditions. Unexpected severe adverse effects related to the planned TDZ dose forced us to use a substantially lower dose of TDZ. This may have compromised the results when compared to the *in vitro* analyses, but these pre-clinical observations are also vital to protect human patients. Thus, these unexpected findings highlight the importance of performing pre-clinical investigations in higher ranked animals such as pigs, which are physiologically and genetically more similar to humans than rodents [34].

Some reports have demonstrated the ability of TDZ to concentrate in macrophages, which is mediated by lysosomal trapping [35–37], and we speculate that infections occurring in macrophage-rich tissue, such as lung, kidney, and liver, may be better suited for systemic treatment with this drug combination. In support of this possibility, Hahn et al. demonstrated reduced bacterial dissemination to the spleen and kidneys [19]. However, further animal pharmacodynamic studies are needed to clarify TDZ’s penetration, uptake, and distribution in various tissues and the impact of infection and inflammation on these parameters.

Another explanation of the *in vitro*/*in vivo* discrepancy is the possibility of a drug-drug interaction between TDZ and DCX. A pharmaco-epidemiologic study has recently confirmed previous reports on DCX’s ability to decrease the effect of liver metabolised drugs such as warfarin [38]. This interaction is most likely mediated through DCX’s activation of the pregnane X receptor, resulting in induction of CYP3A4 [39], which in combination with CYP2D6, significantly contributes to the metabolism of TDZ [40]. Furthermore, a recent report indicates that pigs are fast metabolisers of CYP2D6 substrates [41]. Hence, co-administration of DCX and TDZ in pigs may result in an increased conversion of TDZ into metabolites with potentially lower or no antibacterial activity, but which retain potent neuroleptic effects [42]. This could also explain the observed lack of antimicrobial synergy and the unexpected severe neurologic side effects at dosages which, from human clinical experience, ought to be well-tolerated.

Evidently, this limits the potential of TDZ as an antimicrobial helper compound for systemic treatment. However, TDZ as a helper compound to an antibiotic drug could still be useful for local or topical treatment, for example as part of antimicrobial impregnations of indwelling medical devices or antibacterial ointments against staphylococcal skin infections. We have just recently published a study showing that the combined loading of DCX + TDZ into a polymer hybrid silicone catheter material increased the antibacterial efficiency, although release analyses revealed that this effect was caused by a change in loading kinetics and an enhanced loading capacity of DCX facilitated by the presence of TDZ [23]. Moreover, the purified (-) enantiomer of TDZ, also patented under the name JEK47, apparently exhibits a more favourable toxic profile than its racemate, while maintaining the same antibacterial activity [43]. JEK47 is a promising agent, although *in vivo* evidence of its combined activity with DCX is still lacking.

The idea of enhancing the antimicrobial activity of antibiotics with helper compounds is far from exhausted. Several other prospects of antimicrobial helper compounds, which share chemical structural resemblance with phenothiazines, have been identified and investigated as adjuncts to different antibiotics against *S*. *aureus*. A screening for antimicrobial activity of 1,057 previously approved drugs derived from the WHO and US Food and Drug Administration drug lists, revealed a total of 69 potential non-antibiotic helper compounds that potentiate the antimicrobial activity of a semi-synthetic tetracycline (minocycline) *in vitro* [44]. Thirty-five out of the 69 identified helper compounds demonstrated synergism in combination with minocycline against *S*. *aureus*. Especially, disulfiram (also known as: Antabus) showed a strong synergetic effect on growth inhibition of both MSSA and MRSA strains, including USA300 [44]. Furthermore, chemically designed 2-Phenylquinolines derivatives have proven to be a potent new class of *S*. *aureus* NorA efflux pump inhibitors [45], and studies on β-lactams in combination with cyslabdan, produced by *Streptomyces* sp. K04-0144, have also revealed a considerable reduction in MICs against MRSA [46, 47].

In summary, combining drugs to combat antimicrobial resistance is an intriguing field with a great clinical potential. However, at the same time it adds to biological complexity, both in regard to clinical efficacy and host toxicity, thus *in vivo* studies are demanded. The findings from this study underline the importance of performing *in vivo* studies in large animal models, which to the highest possible degree mimic conditions in humans, before progressing to human clinical trials.

## Supporting information

S1 Table Filled in ARRIVE guidelines checklist. (PDF)
